# Fear and Foxes: An Educational Primer for Use with “Anterior Pituitary Transcriptome Suggests Differences in ACTH Release in Tame and Aggressive Foxes”

**DOI:** 10.1534/genetics.120.303046

**Published:** 2020-03-31

**Authors:** Julie H. Simpson

**Affiliations:** Department of Molecular, Cellular and Developmental Biology and Neuroscience Research Institute, University of California, Santa Barbara, California 93106-9625

**Keywords:** Behavioral genetics, education, gene expression, tameness

## Abstract

The way genes contribute to behavior is complicated. Although there are some single genes with large contributions, most behavioral differences are due to small effects from many interacting genes. This makes it hard to identify the genes that cause behavioral differences. Mutagenesis screens in model organisms, selective breeding experiments in animals, comparisons between related populations with different behaviors, and genome-wide association studies in humans are promising and complementary approaches to understanding the heritable aspects of complex behaviors. To connect genes to behaviors requires measuring behavioral differences, locating correlated genetic changes, determining when, where, and how these candidate genes act, and designing causative confirmatory experiments. This area of research has implications from basic discovery science to human mental health.

IN their article “Anterior Pituitary Transcriptome Suggests Differences in ACTH Release in Tame and Aggressive Foxes,” Hekman *et al.* (2018) use RNA sequencing to measure differences in gene expression in the anterior pituitary (a brain region that secretes hormones) of tame and aggressive foxes to identify candidate genes underlying the selected behavioral differences.

This paper is suitable for a range of teaching contexts. It illustrates the complicated contributions of genes to complex behavioral traits. It provides a venue for discussing the scientific process of question and discovery, hypothesis generation, data collection, validation, analysis, and experimental confirmation. Their methodology, RNA transcriptome sequencing, can be compared to recent genome sequencing analysis on these foxes and to new single-cell RNA profiling methods that have not yet been applied. Candidate genes show differential expression, variations in splicing, and encode proteins with a range of possible functions, providing an opportunity to reinforce concepts from the central dogma (DNA → RNA → protein) and molecular genetics. The RNA sequences obtained from foxes were aligned to the dog genome, which can lead to discussion of canid evolution or the challenges of genome annotation. A recent publication (Lord *et al.* 2019) on how the origin of starting fox population affects potential inferences about domestication allows introduction of concepts from population genetics and how open scientific debate leads to stronger conclusions. Most importantly, this paper is a gateway to a fascinating scientific detective story where the ending is not yet written, showing students that even in the age of rapid DNA sequencing, good experimental design remains critical, and there are many open challenges for the next generation to solve.

## Background

**Figure 1:**
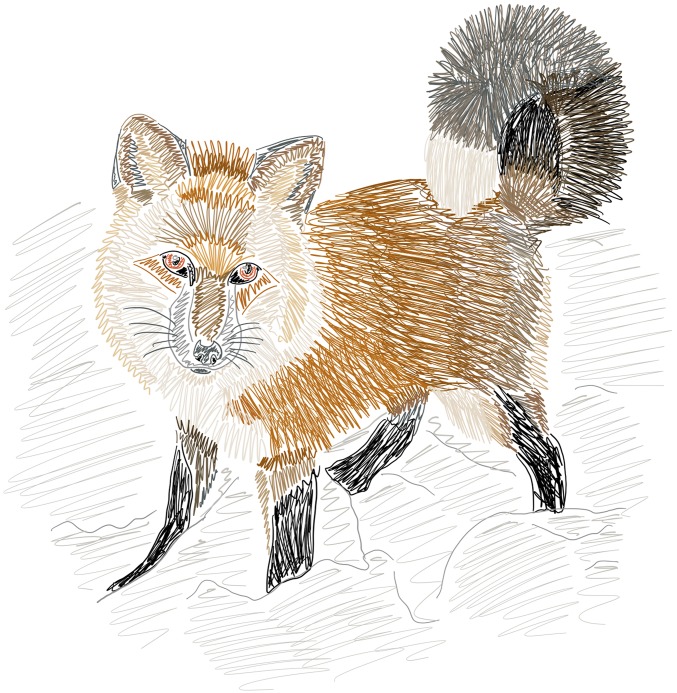
Illustration of a tame fox by Carla Ladd based on a photograph from Irina Pivovarova. See cover image of the March issue of *G3* (https://www.g3journal.org/content/8/3.cover-expansion).

In the 1950s, a fox breeding program was initiated in Siberia (Figure 1). Scientists Belyaev and Trut selected the tamest foxes from fur farms and interbred them. Then they selected the tamest foxes from the next generation and bred those together. Over many generations, they obtained a higher fraction of tame foxes, and the tame foxes were even tamer than their parents. In parallel, they selected the most aggressive foxes and bred those as well. Inbreeding was minimized to maintain effective population sizes so that predominant genetic differences relate to behaviors (Kukekova *et al.* 2004; Trut *et al.* 2012; Johnson *et al.* 2015). Today, >50 generations later, the two populations of foxes are distinctly different, behaviorally and genetically. Researchers recently collected genomic DNA from the tame and aggressive strains, and total messenger RNA (mRNA) from specific areas of their brains. Measurements of circulating hormone levels showed that the tame foxes have less adrenocorticotropic hormone (ACTH) in their blood. Since ACTH is produced in the pituitary, this finding focused attention on that brain area. ACTH is made by enzymatic cleavage of a precursor protein encoded by the proopiomelanocortin (POMC) gene. While POMC is very highly expressed in the pituitary, this gene does not show a difference in expression in the tame and aggressive foxes. Therefore, the authors of this paper searched for differences in expression of other genes that could explain the change in circulating ACTH, such as those regulating its exocytosis (secretion) or degradation.

### Nurture and nature

How much of fox behavior toward humans is controlled by their genes? Some aspects of animal behavior can certainly be modified by training, especially early, reward-based exposure: imagine typical advice for civilizing a new puppy. But there are limits to how much an animal’s behavior can be shaped. Experiments to raise wolf cubs like puppies did not end well (Coppinger and Coppinger 2002; Hall *et al.* 2015). This suggests canids (the genus that includes dogs, wolves, and foxes) have different potential to cohabitate with humans, and that this potential is heritable—controlled by their genes—rather than solely a product of environment (nurture). Although dogs and wolves descended from a common (now extinct) ancestor (Larson *et al.* 2012; Freedman *et al.* 2014; Botigue *et al.* 2017), the current differences in their genomes influence their behavioral repertoires. The search to identify the genetic bases of these behavioral changes is ongoing.

Tameness or aggressiveness in foxes is highly heritable. The fox researchers established that there was a genetic basis for tameness by cross-fostering. They switched kits from tame mothers into the litters of aggressive mothers, and vice versa. (A baby fox is called a kit, pup, or cub; a female fox is a vixen, a male fox—confusingly—is called a dog; and a group of foxes is called a skulk!) They found that the kits’ behavior correlated with that of their genetic parents, rather than that of their foster mother (Trut 1980; Trut 2001; Trut and Dugatkin 2017). This suggests that nurture has less influence on fox kits’ tameness or aggression than does their genetic make-up. Thus, tameness is a highly heritable trait, and it is reasonable to search for the genes that underlie it.

### What is in the genome

The whole fox genome has been sequenced and assembled; comparisons of genomic DNA from tame and aggressive foxes show many differences. To identify the ones that are relevant for the behavioral changes, researchers used techniques from genome analysis (genome-wide association studies, quantitative trait loci, and single nucleotide polymorphisms), as well as tracking allele frequencies in populations and profiling differences in gene expression in the basal forebrain and prefrontal cortex. Candidate “tameness” genes are involved in neuron function, neurotransmitter biosynthesis and release, and neural crest cell migration (Kukekova *et al.* 2018; Wang *et al.* 2018).

### Hypothalamic-pituitary-adrenal axis

**Figure 2:**
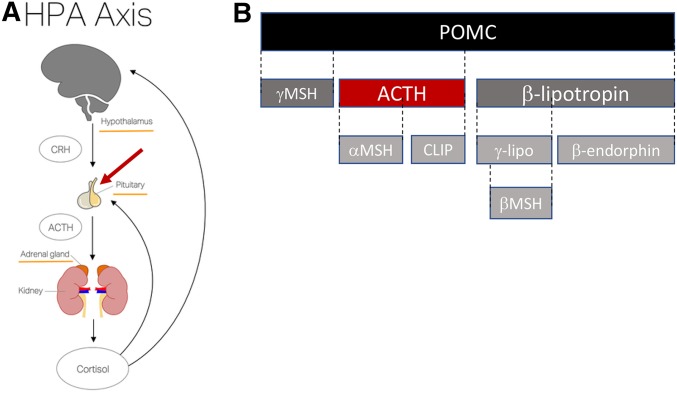
(A) The anatomical pathway and hormonal messengers contributing to the stress response; red arrow indicates anterior pituitary. Adapted from https://mplsimc.com/2017/11/16/tired-burnt-out-need-a-sabbatical-the-science-of-adrenal-fatigue/. (B) Synthesis and degradation of stress hormones. Adapted from https://commons.wikimedia.org/wiki/File:POMC.png.

The researchers in this paper (Hekman *et al.* 2018) chose to focus on gene expression changes in the anterior pituitary because it is a part of the brain that secretes hormones associated with stress. The hypothalamic-pituitary-adrenal axis controls the release of ACTH (see Figure 2A). ACTH stimulates release of glucocorticoids from the adrenal glands (well-known as the source of adrenaline, the “fight-or-flight” trigger). The endocrine system is activated in emergencies and its sensitivity, both likelihood of release of stress hormones and availability of receptors to receive them, can be tuned to set animals’ resistance to stress (Rodrigues *et al.* 2009). Previous experiments comparing tame and aggressive foxes showed differences in circulating stress hormone levels (Gulevich *et al.* 2004), suggesting that these hormones might contribute to their behavior.

This clue, that tame and aggressive foxes showed different amounts of circulating ACTH, led the researcher to look for changes in gene expression in the gland that releases it. There are many candidates. Any gene encoding a protein that alters the production, release, or degradation of ACTH could contribute. ACTH is made in response to corticotropin-releasing hormone (CRH) made by the hypothalamus, generated by cleavage of proopiomelanocortin (POMC; Figure 2B), and degraded into two smaller peptides. The enzymes that produce CRH, the CRH receptors, the enzymes that make ACTH, and even the developmental genes that govern how many ACTH-producing cells are made, were all candidate causes for the reduced stress response in tamer foxes, but the finding that expression of POMC itself is unchanged focused attention on proteins that contribute to the release of ACTH.

## Unpacking the Study

### Measuring differences in gene expression by total RNA sequencing

Although all the cells in the fox have the same chromosomes and genomic DNA, different tissues will express different subsets of their common genomic repertoire. To look for potential changes in gene expression between the tame and aggressive foxes, the authors collected total mRNA from the anterior pituitaries, reverse transcribed it into complementary DNA (cDNA), made that into libraries, and then sequenced. Each of their “reads” is a short sequence that can be matched to a predicted gene on an annotated genome. The presence of a read indicates that the gene is expressed, and the number of reads indicates how highly.

The first analysis shows that 18,940 different genes are expressed in the pituitary, which is a high fraction of the fox’s predicted total of 21,418 protein-coding genes (Kukekova *et al.* 2018). Eleven of the most highly expressed genes (the ones with the most reads) are shown in the authors’ Figure 1. The most highly expressed gene in the pituitary is POMC, and it is not shown on the plot because it is so highly enriched that it disrupts the scale. Other genes are also over-represented in the pituitary, but as we see later, most of these genes are not differentially expressed between tame and aggressive brain samples.

The “normalized gene counts” on the *y*-axis represent the number of copies of a particular gene’s transcript that were present in the collection of cDNA sequences; this helps compare expression levels between genes of different sizes. Each read covers only part of a gene, so without normalization, a big gene with low expression and a small gene with high expression might produce the same number of counts.

Note that the genes are annotated by mapping the fox RNA sequence reads to the dog genome (*Canis familiaris*). This works if the two genes are clear *orthologs* (homologous gene sequences found in different species related by linear descent) but may introduce some counting errors if the dog or the fox have different *paralogs* (similar gene sequences that arrives from a duplication within the species). This possibility is discussed later in the paper in relation to the AVP gene and its close relative OXT. Why did the researchers use the dog genome for alignment if the fox genome has been sequenced, too? Because the dog genome has been more extensively annotated, and the genes and transcripts identified and labeled by homology.

After considering the genes most highly expressed in the pituitary, the authors search for those that are differently expressed between tame and aggressive foxes. In the authors’ Figure 2, the differentially expressed genes (DEGs) are visualized on a volcano plot. A volcano plot is a scatterplot useful for visualizing large data sets where both increases and decreases can occur, and identifying the most significant data points. The magnitude of change is plotted on the *x*-axis and the significance of that change (*P*-value) is plotted on the *y*-axis. (Here, a high *P*-value is more significant, because it is on a negative log scale.) The plot uses a log scale to show fold change rather than absolute transcript differences: this allows genes with both low and high expression levels to be displayed at once, emphasizing their change relative to their own baseline. (For example, *gene a* might show a twofold change of 5–10 arbitrary units, and *gene b* might also show a twofold change but be 200–400 units.) The volcano plot can display both an increase or decrease from the normal value; it is not mirror symmetric. Of the 346 DEGs, 191 are increased in the aggressive foxes (the left side) and 155 are expressed more highly in the tame ones (on the right side). The green dots label the genes that are the most likely to be really important because they are statistically significantly different and because they show a larger fold change.

False discovery rate (FDR) is a method of quantifying the rate of type 1 errors (false positives) in null hypothesis testing when conducting multiple comparisons. Here, the null hypothesis would be that the expression levels of this gene are not different in the two samples, so a false discovery would happen when you call a gene differentially expressed by mistake. FDR-controlling procedures select the acceptable proportion of discoveries that are false (Krzywinski and Altman 2014), obviously hoping to keep them to a minimum. Note that the thresholds for FDR and *P*-values are set by the researchers, based on how stringent or confident they want to be that the genes they follow are biologically different. They could choose lower thresholds and test more candidates, or they could choose more strict ones and only conduct further tests on those with the very highest values.

If you want to explore ways to display different kinds of data like the volcano plot, check out the *Nature Methods* series “Visualizing Data” (http://blogs.nature.com/methagora/2013/07/data-visualization-points-of-view.html), and if you want a more in-depth explanation of the statistics used in biology, see the collected “Points of Significance” series (https://www.nature.com/collections/qghhqm).

Their initial choice of total transcriptome RNA sequencing allowed the authors to get a more global and unbiased view of what genes might contribute to this behavioral difference, with the potential to discover new and unexpected leads. From the analysis shown in the volcano plot, nine candidate genes were selected for validation by quantitative reverse-transcriptase polymerase chain reaction (qRT-PCR). Validation means using an independent and maybe more specific test to address the same research question: it confirms the data, not the interpretation. In this case, the independent test used transcript-specific primers to convert mRNA to cDNA and then amplify the coding portion of each of the nine genes. By extracting samples during the exponential amplification process, researchers can estimate levels of gene expression compared to controls or between samples. Eight genes identified by transcript profiling also showed differential expression in qRT-PCR. The authors could have used an alternative method, *in situ* hybridization, that might also confirm that these genes are expressed in the anterior pituitary but it would probably not indicate expression levels. The RNA transcriptome sequencing and qRT-PCR represent the data collection parts of the paper, and then the authors move on to data analysis.

### Characterizing candidate genes

Some of the DEGs have known functions or have been implicated in biological processes in other research. These possible functions are indicated by Gene Ontology (GO) terms. The GO project (http://geneontology.org) uses a controlled vocabulary to annotate genes based on reports about their function in the scientific literature. For example, the GO term *exocytosis* is defined as “a process of secretion by a cell that results in the release of intracellular molecules (*e.g.* hormones, matrix proteins) contained within a membrane-bounded vesicle” (https://www.ebi.ac.uk/QuickGO/term/GO:0006887). Exocytosis has been used to annotate 92,000 gene or protein sequences. Example genes include *VAMP1* and *synaptotagmin*, encoding components of protein complexes that drive vesicle fusion for neurotransmitter release. Exocytosis is also a key regulated step in hormone secretion. The 2013 Nobel Prize in medicine was awarded for research revealing mechanisms for vesicle trafficking, and a more extensive description of exocytosis can be found here: https://www.nobelprize.org/prizes/medicine/2013/prize-announcement/.

While the value of GO term enrichment depends on the validity of the annotations, the identification of many genes with the same GO terms suggests that those biological processes might be involved in the phenotype. Here, the DEGs in the tame and aggressive fox pituitaries show enrichment for GO terms like Jak-Stat signaling, cell differentiation, POMC regulation, and exocytosis. Table 1 lists some of the GO terms that are over-represented, and Table 2 shows DEGs with shared GO terms.

In another attempt to aggregate different pieces of data to reveal a common cause, the authors used an algorithm to identify groups of genes whose expression changes in the same way. These coregulated networks of genes may have more predictive power than single DEGs (Table 3).

mRNA is formed by splicing introns out of primary transcripts, and some genes undergo alternative splicing, where different exons are included in the final transcript. The tame and aggressive foxes splice some transcripts differently (Table 4). Although alternative splicing occurred in different genes than those identified in the total RNA sequencing experiments, they do share some enriched GO terms.

A subsequent study examined DEGs in the hypothalamus of tame and aggressive foxes as well but identified different candidate genes and GO terms (Rosenfeld *et al.* 2019). This might be expected, since the pituitary and hypothalamus are different brain regions, with their own gene expression profiles and influences on behavior. Further work will be required to determine whether the enriched GO terms have provided useful clues to common processes underlying tameness.

## Extensions and Connections

### A common path to domestication?

Many animals and birds have been bred and selected to live closely with humans in mutually beneficial arrangements. Reduced fear is a common feature of domesticated species (Price 2008) and the hypothalamic-pituitary-adrenal axis in though to play an important role in this (Künzl *et al.* 2003; Ericsson *et al.* 2014). Domesticated animals may exhibit physical and reproductive changes in addition to their behavioral traits. Are there common mechanisms controlling these different aspects of domestication, and are those mechanisms shared among different species? The suite of changes accompanying domestication were originally observed by Charles Darwin, but we still do not have clear answers. While the idea of a “domestication syndrome” is certainly appealing, the measurable hallmarks vary and the evidence for it is problematic (Lord *et al.* 2019).

The farmed fox experiment is often presented as an attempt to reproduce domestication to better understand the process that might have occurred naturally over thousands of years. Selection on tameness may also produce foxes with correlated morphological traits; both kinds of change occur rapidly, in only a few generations. Recent detective work—and mitochondrial DNA sequencing—show that the Russian farmed foxes had ancestors in Canada (Statham *et al.* 2011; Lord *et al.* 2019)! Foxes from Prince Edward Island that had both beautiful fur and the ability to reproduce in captivity contributed to the Russian fur industry, from which the founders for the Belyaev selection experiments were drawn. A founder affect may have contributed to the coappearance of certain physical and behavioral traits.

Several other hypotheses have been proposed for how behavior and appearance could change together. Researchers noticed that the tamer foxes also displayed shorter snouts, curly tails, and white spots. Changes in vocalizations were also reported (Gogoleva *et al.* 2013). In general, the tamer foxes were more kit-like (and dog-like) in appearance. The neural crest hypothesis (Wilkins *et al.* 2014) is one theory that would explain both the behavioral and morphological changes. Neural crest cells form during development and travel along the axis of the body, contributing to different tissues including the brain and spinal cord, ear cartilage, and pigment cells in skin. This hypothesis is also consistent with the rapid acquisition of tameness and puppy-like appearance, and the fact that two animals with intermediate phenotypes can produce offspring with stronger manifestations: there are many different genes contributing to neural crest cell migration and phenotypes can be additive.

Two alternative hypotheses have also been proposed to explain the concordance of behavioral and morphological changes in domestication (Lord *et al.* 2019). In one, changes in thyroid hormone levels contribute to the panoply of pleiotropic effects (Crockford 2006), and in another, neoteny (delays in development or maturation) produces adults with behavioral and physical characteristics more like juveniles (Coppinger *et al.* 1987). A prolonged juvenile phase could also give the foxes longer to adapt to the presence of humans. This is an exciting time because the new genome sequencing and RNA transcript profiling of tame and aggressive fox strains may support one of these hypotheses, or suggest something completely different.

There is some evidence that selection for tameness also alters social cognitive traits, including the unusual ability to communicate with another species—humans! For more extensive discussions of the connection between tameness and cognition in the foxes, see Hare *et al.* (2005), and for an alternative perspective comparing wolves and dog, see Miklósi *et al.* (2003) and Gácsi *et al.* (2009).

The selection of tame and aggressive foxes over 50 years should be considered different from domestication, a process that takes thousands of years. The authors are careful to describe their research as “experimental domestication.” A similar tame/aggressive selection was performed in rats (Heyne *et al.* 2014). In experiments designed to identify gene variants that contribute to behavioral differences, the authors of this study (which include Dr. Trut), bred rats and selected for ones that were tame or aggressive toward humans. After 64 generations of selection, the authors examined quantitative trait loci and sequenced brain mRNA to identify differences in gene expression. The four strongest candidates include a transcription factor and a neurotransmitter transporter but do not overlap with candidates found in the fox study.

An alternative to experimental domestication experiments relies on comparisons between domesticated animals and their wild ancestors. This approach has been taken in guinea pigs, and in chickens, where the pituitary might be important, too. Comparing pituitary transcriptomes from domesticated White Leghorn chicken and wild Red Junglefowl showed differences in gene expression, but not in the same genes as foxes. (Fallahshahroudi *et al.* 2019) The authors did find support for the stress reduction hypothesis: domesticated birds had higher expression of the corticotropin hormone receptor CRHR2, an inhibitor of the stress response pathway, so perhaps different genetic changes affecting the same biological process are at work in different species.

### Intraspecies variability

Dogs, foxes, and modern wolves share a common ancestor. One look at the Westminster Dog Show will demonstrate that there can be enormous physical and behavioral diversity within a species. All these dogs can and have been interbred, so they clearly have reproductively compatible genomes, but they display a huge range of appearance and behavior. Where does this variability come from and why have breeds diverged so quickly? Large initial diversity, strong selective pressure, and few genes with big effects on some traits. In most cases, dog behavior is influenced by many genes (MacLean *et al.* 2019), but occasionally one stands out, such as this paper (vonHoldt *et al.* 2017), where specific changes were linked to hyper-sociability. Dogs are also models for a variety of human conditions: POMC mutations affect obesity in Labradors (Raffan *et al.* 2016), while hypocretin mutations cause narcolepsy in Dobermans (Lin *et al.* 1999). Note the difference between these studies, where single genes have large effect sizes, and the fox behavioral selection experiments, where the behavioral changes seem to be due to many genes. If you want to know more about the dog genome/phenome projects, papers by Ostrander (Kim *et al.* 2018) or Karlsson are good places to start. These websites have links to their projects: https://research.nhgri.nih.gov/dog_genome/ and http://karlssonlab.org/about/our-research/.

The finding that the AVP gene, which encodes vasopressin, is upregulated in the tame foxes is intriguing. Vasopressin has been implicated in vole pair bonding (Winslow *et al.* 1993), monogamy and paternal care in mice (Bendesky *et al.* 2017), and other prosocial behaviors. Perhaps it contributes to tameness in foxes as well. The authors suggest that the differential expression they observe may be artifactual: similarities between the vasopressin and oxytocin gene sequences mean that transcripts of both fox genes may have been assigned as matches to the vasopressin gene in the dog genome. Since the genomic data for both fox and dog is publicly available, and the transcript sequences are provided in the supplementary data for this paper, students could test this possibility as a challenging bioinformatics exercise. Phylogenetic trees and sequence alignments of these genes in foxes, wolves, and dogs could be constructed, illustrating the often-confusing difference between paralogs and orthologs.

### Science in the USSR

It was challenging to do genetic research in the USSR in the Soviet era. Lysenko was very influential and believed that plants or animals could be trained to have desirable properties. His view was similar to Lamarck’s ideas about how giraffes acquired long necks to reach high leaves and that these acquired traits could be inherited by their offspring. These views are discredited now: from the Modern Synthesis of Darwin and Mendel, we know that selection acts on preexisting variation and that traits are inherited as alleles of genes. But in Russia in the 1960s, scientists who conducted experiments that supported different conclusions from Lysenko faced possible exile, imprisonment, and even execution. The Siberian Fox experiments represent an act of scientific courage. For more on the history of genetics in Russia, see Dugatkin and Trut (2017) and this *Genetics* article (Borinskaya *et al.* 2019).

## Connections to Genetics Concepts

From the Genetics Society of America’s CORE concepts and competencies in genetics (https://genetics-gsa.org/education/genetics-learning-framework/), this paper addresses:

Transmission/Patterns of Inheritance
Gene Expression and Regulation
Genetic Variation
Evolution and Population Genetics
Techniques and Methods

## Suggestions for Classroom Use

This paper is an accessible and appealing example of primary literature appropriate for undergraduate and graduate genetics classes. It was selected as one of the *G3* Spotlight articles for 2018. Since it uses several methods (behavioral tests, RNA sequencing, and data analysis), connects to analysis of whole genome sequencing, and addresses fascinating questions (the genetic contribution to behavior and the bases of domestication), it should help students engage with research. The paper generates new questions and hypotheses, rather than settles the debate, which is also useful to illustrate how science usually progresses. As described in the Abstract, there are many different directions to extend outward from this paper depending on the purpose of the class and the interests of its students.

Assigning a short Reading Response due before class promotes a good discussion by ensuring that everyone has read and thought about the paper beforehand. Here is one template with the following prompts:

Name, date, and student identification number.
Paper citation in correct bibliographic format.
State the main hypothesis or question.
Explain a figure.
What was the primary finding or result?
Describe how this finding fits into a larger research field.
Have any papers cited this one?
What did you like and dislike about this paper?
Pose a question to be addressed in class.
What else do you know about this topic?
How does this work fit into the *Genetics* concepts addressed in lecture?

Limiting responses to one page minimizes workload for students and graders, while encouraging practice in writing concise summaries and information synthesis. Writing exercises improve retention of complex ideas (Reynolds *et al.* 2012), peer review can help correct misconceptions (Halim *et al.* 2018), and connections to students’ prior knowledge or interests can promote engagement (Canning *et al.* 2018). In literature seminars or journal clubs that cover multiple papers, reading responses can also serve as notes for the students to remember the primary points of different articles. In managing discussions, it is useful to explicitly consider both the flaws and the contributions of a paper so that conversations do not generate into negative feedback loops. There are good guides for students learning to read scientific literature, such as *Science*’s “How to (seriously) Read a Scientific Paper” (https://www.sciencemag.org/careers/2016/03/how-seriously-read-scientific-paper). See Glass (2010) for a thought-provoking discussion of the differences and relative merits of question-based *vs.* hypothesis-based experiments.

### Discussion questions: molecular genetics

1. Why did the authors sequence the mRNA (the transcriptome) rather than the genomes of the tame and aggressive foxes?
  Note that although which genes are transcribed will differ between cell types, all cells in the fox share a common genome. This is a common misconception reported in genetics courses (Smith and Knight 2012).
  The authors extracted total mRNA from the whole anterior pituitary. If there are multiple cell types present, their different gene expression profiles would be averaged. A new alternative method is single-cell RNA sequencing, where the partial transcriptomes from many individual cells can be obtained (Macosko *et al.* 2015). What might be the advantages and disadvantages of using this method here?
2. How many foxes and cells were sequenced? How big were the tame and aggressive populations? Is sample size important?
3. If we examined the genomic data, would we expect to find candidate mutations in regulatory DNA (enhancers, repressors, promotors) or protein coding regions?
4. How do you get differences in gene expression levels (regulatory mutations, codon usage, epigenetic effects…)?
5. If we have the genomes and the transcriptomes, what are we missing?
  Consider epigenetic regulation (chromatin landscapes), post-translational modifications (protein phosphorylation, degradation, etc.), cell-specific effects.
6. Where did the mutations or variant alleles come from?
  Note that the origin of variation/mutations and whether mutations are intrinsically deleterious/beneficial are also often difficult concepts for genetics students (Smith and Knight 2012).
  The gene variants (alleles) were probably preexisting in the starting population. This may also explain why foxes keep getting tamer in subsequent generations: different alleles in different genes contributing to tameness were brought together. Tameness is a multigenic trait with each gene variant contributing a small effect.

### Discussion questions: evolution and population genetics

7. Turbo-taming: By the sixth generation, the fox breeders had some significantly tamer foxes. How can a trait change so quickly?
  Many genetic pathways and many different genes can contribute to this behavior; strong selective pressure.
8. How does evidence that these fox breeding experiments were probably started with a limited number of founders brought from Canadian farms affect the potential and conclusions of this research?
  The mapping between changes in gene expression or genome sequence and measurable behavior differences is unaffected, but the bottlenecks and founder effects may have reduced the starting diversity upon which the strong experimental selection can act. If the founders were partially tame to begin with, the speed of behavioral change is now harder to measure: the aggressive foxes may actually show how quickly tameness can be undone. And it remains difficult to eliminate human experimenter bias in selection to ensure that behavior only, not appearance, contributed to the choice of breeding pairs.
  Is there a way to assess population sizes from the genetic data?
9. In a paper comparing genomic DNA of tame and aggressive rats (Heyne *et al.* 2014), the authors say, “Many causative alleles were not driven to fixation by the selection.” What does this mean? Are there different challenges when comparing genomes between closely related (same species) and distantly related (different species) animals? What about comparing the genomes of dogs and wolves? Both practical experimental issues and data analysis problems can be considered.

### Discussion questions: methods, techniques, and experimental design

An instructor might ask students to consider how they would approach identifying the cause of tameness and aggressiveness in the foxes before introducing the paper.

10. What are the right controls? What mRNA transcriptomes should be compared?
  Aggressive: unselected: tame. There will certainly be differences among the tame animals, but places where all tame animals have one variant and all aggressive animals have a different one become the most likely candidates.
  For the GO term analysis, scrambled samples containing both tame and aggressive data could be generated synthetically and assessed for spurious or artifactual enrichment.
11. Once the authors identify genes and alleles they hypothesize might contribute to tameness, what additional steps could they take to test them?
  Discuss the difference between correlation and causation, and proximate causes *vs.* secondary consequences: If you observe a change in gene expression levels or splicing, how can you tell whether this change is at the top of the cascade or a downstream, secondary consequence of some more causative change? What if being tamer makes foxes sleepy and then all the other changes in gene expression you see are actually due to less exercise? What if these gene changes just make foxes stupid? Or smarter?
  There are challenges to working in atypical model organisms and on traits such as behavior, which are likely to have many contributing genes with small effect sizes. One experimental path would be to make the analogous mutations in mice and measure their behavioral effects. This approach was taken in analysis of horse gaits: the genes were identified by comparing genomes of horses with unusual gaits and then functionally tested in mice.(Andersson *et al.* 2012).
  If you were a new graduate student in this laboratory, which of these gene candidates (or GO processes) would you pick to work on? Why?
  What does the GRK7 transcript encode? How many members of this protein family are there in foxes or dogs?
12. Tinbergen’s four questions: There are different levels at which behavior can be analyzed (Bateson and Laland 2013). What levels do you think are represented in this work?
  - Proximate: causation (mechanism)
  - Proximate: ontogeny (development)
  - Ultimate: evolution (phylogeny)
  - Ultimate: survival value (adaptive significance or current utility).

This paper can lead to an expanded discussion of genetic contributions to behavior, from laboratory experiments in model organisms (Benzer 1973; Baker *et al.* 2001) to citizen science efforts using existing variation in dogs (MacLean *et al.* 2019). Comparisons between identical and fraternal twins, using genome-wide association studies and quantitative trait loci analyses, examine the contributions of genes to human behaviors from psychological disorders (schizophrenia and autism) to educational attainment (Lee *et al.* 2018) and sexual preferences (Ganna *et al.* 2019). It is particularly important to consider that while genes certainly influence behavior, it is usually many genes with small effect sizes acting together to control a change.

## Perspective

This paper is a hypothesis generator: the authors get some ideas about what processes might be worth further investigation, but there is no smoking gun. The causes of tameness and aggression might not be in the pituitary at all, or might require lots of small changes in gene expression, or protein function through post-translational effects. In the parlance of the central dogma, DNA → RNA → protein, this paper sits at RNA. Further research could explore DNA contributions with whole genome comparisons (Kukekova *et al.* 2018; Wang *et al.* 2018), single-cell RNA sequencing, or proteomic analyses. Even with all of these modern experimental methods, it is not at all easy to sort out the underlying causes of complex behavioral traits!

## References

[bib1] Andersson L. S., Larhammar M., Memic F., Wootz H., Schwochow D., 2012 Mutations in DMRT3 affect locomotion in horses and spinal circuit function in mice. Nature 488: 642–646. 10.1038/nature11399 22932389 PMC3523687

[bib2] Baker B. S., Taylor B. J., and Hall J. C., 2001 Are complex behaviors specified by dedicated regulatory genes? Reasoning from Drosophila. Cell 105: 13–24. 10.1016/S0092-8674(01)00293-8 11300999

[bib3] Bateson P., and Laland K. N., 2013 Tinbergen’s four questions: an appreciation and an update. Trends Ecol. Evol. 28: 712–718. 10.1016/j.tree.2013.09.013 24144467

[bib4] Bendesky A., Kwon Y. M., Lassance J. M., Lewarch C. L., Yao S. Q., 2017 The genetic basis of parental care evolution in monogamous mice. Nature 544: 434–439. 10.1038/nature22074 28424518 PMC5600873

[bib5] Benzer S., 1973 Genetic dissection of behavior. Sci. Am. 229: 24–37. 10.1038/scientificamerican1273-24 4202065

[bib6] Borinskaya S. A., Ermolaev A. I., and Kolchinsky E. I., 2019 Lysenkoism against genetics: the meeting of the lenin all-union academy of agricultural sciences of august 1948, its background, causes, and aftermath. Genetics 212: 1–12. 10.1534/genetics.118.301413 31053614 PMC6499510

[bib7] Botigué L. R., Song S., Scheu A., Gopalan S., Pendleton A. L., 2017 Ancient European dog genomes reveal continuity since the Early Neolithic. Nat. Commun. 8: 16082 10.1038/ncomms16082 28719574 PMC5520058

[bib8] Canning E. A., Harackiewicz J. M., Priniski S. J., Hecht C. A., Tibbetts Y., 2018 Improving performance and retention in introductory biology with a utility-value intervention. J. Educ. Psychol. 110: 834–849. 10.1037/edu0000244 30294006 PMC6168083

[bib9] Coppinger R. P., and Coppinger L., 2002 *Dogs: A New Understanding of Canine Origin*, *Behavior*, *and Evolution*, University of Chicago Press, Chicago, IL.

[bib10] Coppinger R., Glendinning J., Torop E., Matthay C., Sutherland M., 1987 Degree of behavioral neoteny differentiates canid polymorphs. Ethology 75: 89–108. 10.1111/j.1439-0310.1987.tb00645.x

[bib11] Crockford S. J., 2006 Rhythms of Life: Thyroid Hormone & the Origin of Species, Trafford, Victoria, BC.

[bib12] Dugatkin L. A., and Trut L. N., 2017 How to Tame a Fox (and Build a Dog): Visionary Scientists and a Siberian Tale of Jump-Started Evolution, The University of Chicago Press, Chicago 10.7208/chicago/9780226444215.001.0001

[bib13] Ericsson M., Fallahsharoudi A., Bergquist J., Kushnir M. M., and Jensen P., 2014 Domestication effects on behavioural and hormonal responses to acute stress in chickens. Physiol. Behav. 133: 161–169. 10.1016/j.physbeh.2014.05.024 24878317

[bib14] Fallahshahroudi A., Lotvedt P., Belteky J., Altimiras J., and Jensen P., 2019 Changes in pituitary gene expression may underlie multiple domesticated traits in chickens. Heredity 122: 195–204. 10.1038/s41437-018-0092-z 29789643 PMC6327055

[bib15] Freedman A. H., Gronau I., Schweizer R. M., Ortega-Del Vecchyo D., Han E., 2014 Genome sequencing highlights the dynamic early history of dogs. PLoS Genet. 10: e1004016 (erratum: PLoS Genet. 10: e1004631). 10.1371/journal.pgen.1004016 24453982 PMC3894170

[bib16] Gácsi M., Gyori B., Viranyi Z., Kubinyi E., Range F., 2009 Explaining dog wolf differences in utilizing human pointing gestures: selection for synergistic shifts in the development of some social skills. PLoS One 4: e6584 (erratum: PLoS One 4(9)). 10.1371/journal.pone.0006584 19714197 PMC2719091

[bib17] Ganna A., Verweij K. J. H., Nivard M. G., Maier R., Wedow R., 2019 Large-scale GWAS reveals insights into the genetic architecture of same-sex sexual behavior. Science 365: eaat7693. 10.1126/science.aat7693 PMC7082777 31467194

[bib18] Glass D. J., 2010 A critique of the hypothesis, and a defense of the question, as a framework for experimentation. Clin. Chem. 56: 1080–1085. 10.1373/clinchem.2010.144477 20511448

[bib19] Gogoleva S. S., Volodin I. A., Volodina E. V., Kharlamova A. V., and Trut L. N., 2013 Effects of selection for behavior, human approach mode and sex on vocalization in silver fox. J. Ethol. 31: 95–100. 10.1007/s10164-012-0353-x 23525128 PMC3601802

[bib20] Gulevich R. G., Oskina I. N., Shikhevich S. G., Fedorova E. V., and Trut L. N., 2004 Effect of selection for behavior on pituitary-adrenal axis and proopiomelanocortin gene expression in silver foxes (Vulpes vulpes). Physiol. Behav. 82: 513–518. 10.1016/j.physbeh.2004.04.062 15276817

[bib21] Halim A. S., Finkenstaedt-Quinn S. A., Olsen L. J., Gere A. R., and Shultz G. V., 2018 Identifying and remediating student misconceptions in introductory biology via writing-to-learn assignments and peer review. CBE Life Sci. Educ. 17: ar28 10.1187/cbe.17-10-0212 29749850 PMC5998326

[bib22] Hall N. J., Lord K., Arnold A. M., Wynne C. D., and Udell M. A., 2015 Assessment of attachment behaviour to human caregivers in wolf pups (Canis lupus lupus). Behav. Processes 110: 15–21. 10.1016/j.beproc.2014.11.005 25447510

[bib23] Hare B., Plyusnina I., Ignacio N., Schepina O., Stepika A., 2005 Social cognitive evolution in captive foxes is a correlated by-product of experimental domestication. Curr. Biol. 15: 226–230. 10.1016/j.cub.2005.01.040 15694305

[bib24] Hekman J. P., Johnson J. L., Edwards W., Vladimirova A. V., Gulevich R. G., 2018 Anterior pituitary transcriptome suggests differences in ACTH release in tame and aggressive foxes. G3 (Bethesda) 8: 859–873. 10.1534/g3.117.300508 29378821 PMC5844307

[bib25] Heyne H. O., Lautenschlager S., Nelson R., Besnier F., Rotival M., 2014 Genetic influences on brain gene expression in rats selected for tameness and aggression. Genetics 198: 1277–1290. 10.1534/genetics.114.168948 25189874 PMC4224166

[bib26] Johnson J. L., Wittgenstein H., Mitchell S. E., Hyma K. E., Temnykh S. V., 2015 Genotyping-by-sequencing (GBS) detects genetic structure and confirms behavioral QTL in tame and aggressive foxes (Vulpes vulpes). PLoS One 10: e0127013 10.1371/journal.pone.0127013 26061395 PMC4465646

[bib27] Kim J., Williams F. J., Dreger D. L., Plassais J., Davis B. W., 2018 Genetic selection of athletic success in sport-hunting dogs. Proc. Natl. Acad. Sci. USA 115: E7212–E7221. 10.1073/pnas.1800455115 29970415 PMC6065024

[bib28] Krzywinski M., and Altman N., 2014 Comparing samples-part II. Nat. Methods 11: 355–356. 10.1038/nmeth.2900

[bib29] Kukekova A. V., Trut L. N., Oskina I. N., Kharlamova A. V., Shikhevich S. G., 2004 A marker set for construction of a genetic map of the silver fox (Vulpes vulpes). J. Hered. 95: 185–194. 10.1093/jhered/esh033 15220384

[bib30] Kukekova A. V., Johnson J. L., Xiang X., Feng S., Liu S., 2018 Red fox genome assembly identifies genomic regions associated with tame and aggressive behaviours. Nat. Ecol. Evol. 2: 1479–1491 (erratum: Nat. Ecol. Evol. 2: 1517). 10.1038/s41559-018-0611-6 30082739 PMC6663081

[bib31] Künzl C., Kaiser S., Meier E., and Sachser N., 2003 Is a wild mammal kept and reared in captivity still a wild animal? Horm. Behav. 43: 187–196. 10.1016/S0018-506X(02)00017-X 12614649

[bib32] Larson G., Karlsson E. K., Perri A., Webster M. T., Ho S. Y., 2012 Rethinking dog domestication by integrating genetics, archeology, and biogeography. Proc. Natl. Acad. Sci. USA 109: 8878–8883. 10.1073/pnas.1203005109 22615366 PMC3384140

[bib33] Lee J. J., Wedow R., Okbay A., Kong E., Maghzian O., 2018 Gene discovery and polygenic prediction from a genome-wide association study of educational attainment in 1.1 million individuals. Nat. Genet. 50: 1112–1121. 10.1038/s41588-018-0147-3 30038396 PMC6393768

[bib34] Lin L., Faraco J., Li R., Kadotani H., Rogers W., 1999 The sleep disorder canine narcolepsy is caused by a mutation in the hypocretin (orexin) receptor 2 gene. Cell 98: 365–376. 10.1016/S0092-8674(00)81965-0 10458611

[bib35] Lord K. A., Larson G., Coppinger R. P., and Karlsson E. K., 2019 The history of farm foxes undermines the animal domestication syndrome. Trends Ecol. Evol. 35: 125–136. 10.1016/j.tree.2019.10.011 31810775

[bib36] MacLean E. L., Snyder-Mackler N., vonHoldt B. M., and Serpell J. A., 2019 Highly heritable and functionally relevant breed differences in dog behaviour. Proc. R. Soc. B. 286: 20190716. 10.1098/rspb.2019.0716 PMC6790757 31575369

[bib37] Macosko E. Z., Basu A., Satija R., Nemesh J., Shekhar K., 2015 Highly parallel genome-wide expression profiling of individual cells using nanoliter droplets. Cell 161: 1202–1214. 10.1016/j.cell.2015.05.002 26000488 PMC4481139

[bib38] Miklósi A., Kubinyi E., Topal J., Gacsi M., Viranyi Z., 2003 A simple reason for a big difference: wolves do not look back at humans, but dogs do. Curr. Biol. 13: 763–766. 10.1016/S0960-9822(03)00263-X 12725735

[bib39] Price E. O., 2008 Principles and Applications of Domestic Animal Behavior: An Introductory Text, CAB International, Oxfordshire, England.

[bib40] Raffan E., Dennis R. J., O’Donovan C. J., Becker J. M., Scott R. A., 2016 A deletion in the canine POMC gene is associated with weight and appetite in obesity-prone labrador retriever dogs. Cell Metab. 23: 893–900. 10.1016/j.cmet.2016.04.012 27157046 PMC4873617

[bib41] Reynolds J. A., Thaiss C., Katkin W., and Thompson R. J. Jr, 2012 Writing-to-learn in undergraduate science education: a community-based, conceptually driven approach. CBE Life Sci. Educ. 11: 17–25. 10.1187/cbe.11-08-0064 22383613 PMC3292059

[bib42] Rodrigues S. M., LeDoux J. E., and Sapolsky R. M., 2009 The influence of stress hormones on fear circuitry. Annu. Rev. Neurosci. 32: 289–313. 10.1146/annurev.neuro.051508.135620 19400714

[bib43] Rosenfeld C. S., Hekman J. P., Johnson J. L., Lyu Z., Ortega M. T., 2019 Hypothalamic transcriptome of tame and aggressive silver foxes (Vulpes vulpes) identifies gene expression differences shared across brain regions. Genes Brain Behav. 19: e12614 10.1111/gbb.12614 31605445 PMC7385667

[bib44] Smith M. K., and Knight J. K., 2012 Using the Genetics Concept Assessment to document persistent conceptual difficulties in undergraduate genetics courses. Genetics 191: 21–32. 10.1534/genetics.111.137810 22367036 PMC3338261

[bib45] Statham M. J., Trut L. N., Sacks B. N., Kharlamova A. V., Oskina I. N., 2011 On the origin of a domesticated species: identifying the parent population of Russian silver foxes (Vulpes vulpes). Biol. J. Linn. Soc. Lond. 103: 168–175. 10.1111/j.1095-8312.2011.01629.x 21625363 PMC3101803

[bib46] Trut L., 1980 The genetics and phenogenetics of domestic behavior, pp. 123–136 in *Proceedings of the XIV International Congress of Genetics* MIR Publishers Moscow, Moscow.

[bib47] Trut L., 2001 Experimental studies of early canid domestication, pp. 15–41 in *The Genetics of the Dog,* edited by A. Ruvinsky and J. Sampson. CAB International, Oxfordshire, England 10.1079/9780851995205.0015

[bib48] Trut L., and Dugatkin L. A., 2017 How to build a dog. Sci. Am. 316: 69–73. 10.1038/scientificamerican0517-68 28437410

[bib49] Trut L., Oskina I. N., and Kharlamova A. V., 2012 Experimental studies of early canid domestication, pp. 12–37 in The Genetics of the Dog, edited by Ostrander E. A., and Ruvinsky A. CAB International, Oxfordshire, England 10.1079/9781845939403.0012

[bib50] vonHoldt B. M., Shuldiner E., Koch I. J., Kartzinel R. Y., Hogan A., 2017 Structural variants in genes associated with human Williams-Beuren syndrome underlie stereotypical hypersociability in domestic dogs. Sci. Adv. 3: e1700398 10.1126/sciadv.1700398 28776031 PMC5517105

[bib51] Wang X., Pipes L., Trut L. N., Herbeck Y., Vladimirova A. V., 2018 Genomic responses to selection for tame/aggressive behaviors in the silver fox (Vulpes vulpes). Proc. Natl. Acad. Sci. USA 115: 10398–10403. 10.1073/pnas.1800889115 30228118 PMC6187196

[bib52] Wilkins A. S., Wrangham R. W., and Fitch W. T., 2014 The “domestication syndrome” in mammals: a unified explanation based on neural crest cell behavior and genetics. Genetics 197: 795–808. 10.1534/genetics.114.165423 25024034 PMC4096361

[bib53] Winslow J. T., Hastings N., Carter C. S., Harbaugh C. R., and Insel T. R., 1993 A role for central vasopressin in pair bonding in monogamous prairie voles. Nature 365: 545–548. 10.1038/365545a0 8413608

